# Targeting the Initiator Protease of the Classical Pathway of Complement Using Fragment-Based Drug Discovery

**DOI:** 10.3390/molecules25174016

**Published:** 2020-09-03

**Authors:** Blake R. Rushing, Denise L. Rohlik, Sourav Roy, D. Andrew Skaff, Brandon L. Garcia

**Affiliations:** 1 Department of Microbiology and Immunology, Brody School of Medicine, East Carolina University, Greenville, NC 27858, USA; blake_rushing@unc.edu (B.R.R.); rohlikd18@students.ecu.edu (D.L.R.); roys19@ecu.edu (S.R.); 2 Eir Pharmaceuticals, LLC, Olathe, KS 66061, USA; skaff@eirpharmaceuticals.com

**Keywords:** complement inhibitors, fragment-based drug discovery, surface plasmon resonance

## Abstract

The initiating protease of the complement classical pathway, C1r, represents an upstream and pathway-specific intervention point for complement-related autoimmune and inflammatory diseases. Yet, C1r-targeted therapeutic development is currently underrepresented relative to other complement targets. In this study, we developed a fragment-based drug discovery approach using surface plasmon resonance (SPR) and molecular modeling to identify and characterize novel C1r-binding small-molecule fragments. SPR was used to screen a 2000-compound fragment library for binding to human C1r. This led to the identification of 24 compounds that bound C1r with equilibrium dissociation constants ranging between 160–1700 µM. Two fragments, termed CMP-1611 and CMP-1696, directly inhibited classical pathway-specific complement activation in a dose-dependent manner. CMP-1611 was selective for classical pathway inhibition, while CMP-1696 also blocked the lectin pathway but not the alternative pathway. Direct binding experiments mapped the CMP-1696 binding site to the serine protease domain of C1r and molecular docking and molecular dynamics studies, combined with C1r autoactivation assays, suggest that CMP-1696 binds within the C1r active site. The group of structurally distinct fragments identified here, along with the structure–activity relationship profiling of two lead fragments, form the basis for future development of novel high-affinity C1r-binding, classical pathway-specific, small-molecule complement inhibitors.

## 1. Introduction

**Figure 1:**
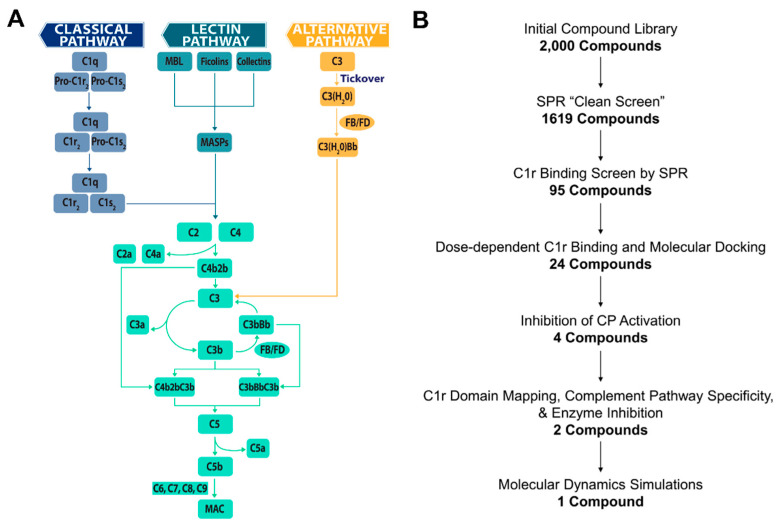
(**A**) Complement is activated by three canonical pathways known as the classical pathway (CP), lectin pathway (LP), or alternative pathway (AP). Activation of the classical pathway is controlled by the C1 complex (i.e., C1qC1r_2_C1s_2_). The pattern recognition protein C1q binds to target surfaces resulting in the autoactivation of the zymogen C1r proteases (shown here as ‘Pro-C1r’) into C1r enzymes, which then proteolytically cleave and activate C1s within the C1 complex. The lectin pathway is activated by lectin pathway-specific pattern recognition proteins in complex with mannan-binding associated serine proteases (MASPs), while the alternative pathway is constitutively activated at low levels by a spontaneous hydrolytic event known as tick-over. Both the classical and lectin pathways converge at the cleavage of C2 and C4 to generate the classical/lectin pathway C3 convertases, C4b2b. Alternative pathway activation results in the formation of C3 convertases in the form of C3bBb. C3 convertases cleave the central molecule of the cascade, C3, into C3a and C3b, resulting in an amplification loop that produces increasing quantities of surface bound C3b. At high surface concentrations of C3b, C3 convertases bind an additional C3b molecule, resulting in a switch of substrate specificity to C5. Cleavage of C5 by these C5 convertases (i.e., C4b2bC3b and C3bBbC3b) results in the release of the anaphylatoxin C5a and the formation of the pore-like lytic structure called the membrane attack complex (i.e., C5b–C9). (**B**) Fragment-based drug discovery schematic.

The complement system is a primary arm of innate immunity involved in recognizing and eliminating infectious agents, marking and removing cellular debris, maintaining homeostasis, and triggering inflammation [1]. Complement activation occurs through one of three canonical pathways known as the classical pathway, lectin pathway, or alternative pathway (Figure 1A) [2,3,4]. Whereas the alternative pathway is constitutively activated via a spontaneous hydrolytic process known as ‘tick-over’, the lectin pathway and classical pathway are defined by the relative activities of pathway-associated pattern recognition proteins. Independent of the initiating event, all three pathways lead to the activation of the central molecule of the cascade, complement component C3. Cleavage of C3 into C3a and C3b by enzymatic complexes, known as C3 convertases (i.e., C3bBb and C4b2b), results in complement amplification on, and opsonization of, target surfaces. C3 activation also initiates the distal reactions of the complement cascade through cleavage of C5 into C5a and C5b by C5 convertases (i.e., C3bBbC3b and C4b2bC3b). Ultimately, activation of the complement cascade leads to the recruitment of professional phagocytes via release of powerful chemotactic polypeptides (i.e., C3a and C5a), opsonization of surfaces and particles near the site of activation with various complement protein fragments (i.e., C3b, iC3b, C3dg, and C3d), and the formation of a pore-like lytic structure known as the membrane attack complex (MAC) that directly lyses susceptible target membranes (i.e., C5b-9) (Figure 1A) [2,3].

Despite its functions in immune surveillance and homeostasis, complement is also implicated in the pathophysiology of a multitude of autoimmune diseases, inflammatory conditions, and degenerative disorders [5,6,7,8,9,10]. Complement-related diseases cover the spectrum of acute to chronic conditions and are associated with both local and systemic disorders [8]. The relationship of complement to human diseases is complex and disease-specific. Complement-related disorders—like C3 glomerulopathy (C3G), paroxysmal nocturnal hemoglobinuria (PNH), and atypical hemolytic uremic syndrome (aHUS)—are attributed to inappropriately regulated complement activation and are often strongly associated with mutations in key complement regulatory proteins, like factor H, CD46, CD55, and/or CD59 [7,8,11]. Even in the absence of underlying genetic causes, dysregulation of complement can cause inappropriate activation leading to adverse effects, as is the case in ischemia/reperfusion injury or transplant rejection [8]. In other diseases, including neurological disorders like Alzheimer’s disease [12,13,14] or thrombotic conditions like heparin-induced thrombocytopenia (HIT) [15,16], the underlying mechanisms of complement involvement are only now becoming more clear. Nevertheless, mounting evidence for the involvement of complement in many human diseases has spurred a resurgence of activity in the field of complement-directed therapeutics [8].

The developmental landscape of anti-complement therapies is now robust and includes many drugs in preclinical and clinical stages [7,8,17]. Two complement drugs, C1 esterase inhibitor (C1-INH) and the anti-C5 antibody drug eculizumab/Soliris, have reached market, although C1-INH is FDA-approved for an indication not primarily mediated by complement (hereditary angioedema (HAE)). Eculizumab/Soliris has now gained FDA-approval for use in in PNH, aHUS, and refractory generalized myasthenia gravis (gMG) [7,17,18]. Importantly, both drugs are also being evaluated in clinical trials for other complement-related diseases [7,17,18]. Eculizumab and C1-INH are joined by dozens of new complement-directed drugs that include small molecules, antibodies, biologics, peptides, and nucleotide-based therapies for the treatment of complement-related diseases, such as aHUS, C3G, PNH, antibody-mediated rejection, IgA nephropathy, age-related macular degeneration (AMD), and many others [7,17,18].

Among the proteins that comprise the complement system are a small set of serine proteases with highly restricted substrate specificity. These include the initiating proteases of the alternative pathway (i.e., MASP-3/factor D), lectin pathway (i.e., MASP-1), and the classical pathway (i.e., C1r). Due to their far upstream position, initiating proteases represent a promising complement intervention point. Targeting complement at the level of MASP-1 or C1r provides a second advantage due to the potential of providing a pathway-specific blockade. While factor D-mediated formation of alternative pathway C3 convertases (i.e., C3bBb) amplifies all three pathways (Figure 1A), MASP-1 and C1r activities are specific for the lectin or classical pathways, respectively. Thus, selective inhibition of MASP-1 or C1r may be ideal in a disease setting where pathophysiological activation of complement is specific to the lectin pathway or classical pathway, as it would theoretically leave two activation pathways available for immune surveillance. Furthermore, proteases are regarded as highly druggable targets with some estimates placing them as targets in 5–10% of all drug development [19]. Not surprisingly, proteases of the complement system have garnered attention as therapeutic targets [7,17,18,20]. However, to date, there has been no significant pharmaceutical development of inhibitors specifically directed towards MASP-1 or C1r. In this regard, C1r is particularly attractive as inappropriate classical pathway activation is implicated in an increasing number of diseases, including HIT [15], neuromyelitis optica [21], bullous pemphigoid [22,23], and autoimmune hemolytic anemias [24], among others. The classical pathway has also been shown to play causal roles in mouse models of Alzheimer’s disease [12] and has been genetically linked to other neurological disorders, such as schizophrenia [25].

A common challenge for the development of drugs that target serine proteases like C1r—especially with small molecules—is addressing specificity. Interestingly, the role of C1r as the initiator protease of the classical pathway requires the molecular context of the C1 complex. Furthermore, its physiological autoactivation and subsequent catalytic activity on C1s proenzyme involves a precise set of coordinated intramolecular events within the C1 complex (i.e., C1qC1r_2_C1s_2_) [26,27,28]. For example, C1r is autoactivated only when C1q binds to immune complexes or activating non-antibody ligands, which is then followed by C1r cleavage of C1s into its active form (Figure 1A). C1r is positioned in the C1 complex via intermolecular contacts with both C1q and C1s [29,30] and disruption or displacement of C1r from C1 has been described as a complement evasion strategy employed by human microbial pathogens [31]. Therefore, we hypothesized that along with orthosteric C1r-binding inhibitors, small molecules that target C1r at sites required for C1 complex stability could lead to the development of highly selective classical pathway inhibitors. 

With these considerations in mind and with the long-term goal of producing a highly specific small-molecule C1 inhibitor, we report here the results of a C1r-targeted fragment-based drug discovery (FBDD) campaign (Figure 1B). FBDD employs compound screening libraries of very low molecular weight small-molecule fragments ≤ 300 Da [32,33,34]. Fragment screening offers vastly increased hit rates and allows more efficient sampling of chemical space relative to traditional high throughput screening of large elaborated compound libraries [32,33,34]. While fragment-based hits are most often characterized by low-affinity binders with equilibrium dissociation constants (*K*_D_) and half-maximal inhibitory concentrations (IC_50_) ≥ 100 µM, their small starting size gives greater flexibility during compound optimization stages [32,33,34]. Our FBDD campaign ultimately yielded 24 small-molecule fragments that reversibly bind full-length human C1r with affinities that ranged from 160 µM to 1700 µM. Four of these C1r-binding fragments had direct inhibitory activity in serum-based classical pathway activation assays. We selected two lead fragments, termed CMP-1611 and CMP-1696, and carried out detailed structure-function analysis which showed that these two compounds differ in their selectivity profile and possibly in their mechanisms of action. The discovery here of structurally distinct classes of C1r-binding fragments represents a significant step forward in the development of novel small-molecule inhibitors of the classical pathway.

## 2. Results

### 2.1. Small-Molecule Fragment Library Design

This study began with the design and acquisition of a custom-selected, commercially synthesized, 2000-compound small-molecule library (ChemDiv Inc., San Diego, CA, USA). The library was composed of five subsets: (i) 250 ‘two-dimensional fragments’ (2D-FL: CMP-1 to CMP-250), (ii) 250 ‘three-dimensional fragments’ (3D-FL: CMP-251 to CMP-500), (iii) 250 ‘natural product scaffolds’ (NPB: CMP-501 to CMP-750), (iv) 250 ‘serine–protease inhibitor’ compounds (SPI: CMP-751 to CMP-1000), and (v) 1000 ‘protein–protein interaction inhibitor’ compounds (PPI: CMP-1001 to 2000). Compounds originating from the 2D-FL subset represent traditional small-molecule fragments ranging in size between 99–330 Da, while the 3D-FL library were fragment-sized compounds (109–372 Da) with increased three-dimensional character, as judged by increased sp^3^ hybridized carbons [35]. Because C1r is a serine protease, we also acquired a small SPI library that consisted of both small molecules and fragments (164–575 Da) with scaffold similarity to known protease inhibitors. To support the potential discovery of protein–protein interaction inhibitors that may inhibit C1r directly or disrupt C1r’s position within the C1 complex, we also selected a NPB library of small molecules and fragments (221–568 Da), which included compounds with scaffolds that share similarity to natural product-derived compounds. For the same reasons, we used a PPI library (138–580 Da) which included compounds inspired by the chemical features of known protein–protein interaction inhibitors. Collectively, these sub-libraries encompassed diverse chemical scaffolds, functional groups, stereochemistry, conformers, and substituent moieties.

### 2.2. Initial Library Screening of C1r-Binding by Surface Plasmon Resonance

**Figure 2:**
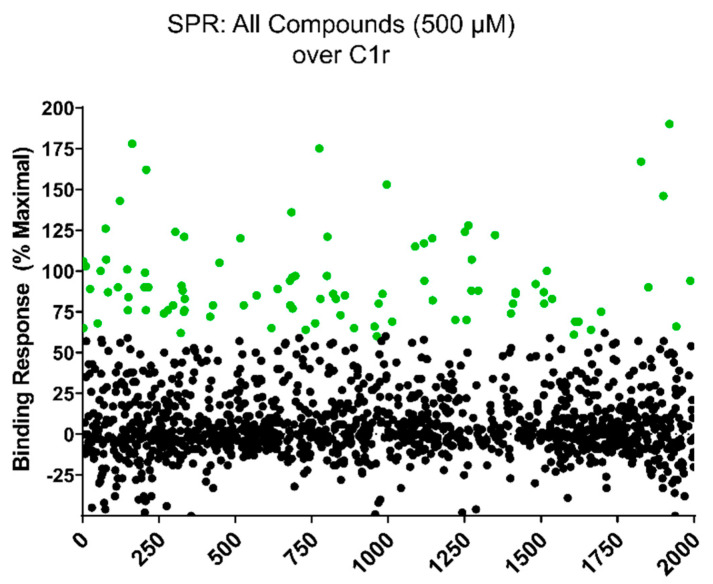
Direct binding of compounds to full-length C1r by SPR. A 2000-compound library was screened at 500 µM final compound concentration for solubility in SPR buffer and for non-specific binding to a blank sensor chip surface (i.e., ‘clean screen’). A total of 1619 compounds were soluble and exhibited low non-specific binding capacity in our SPR assay system. The ability of each of these compounds to bind directly to C1r was measured by injecting a 500 µM final compound concentration over immobilized full-length C1r. A molecular weight corrected theoretical maximal binding response (R_max_) for each compound was calculated and compounds that exhibited superstoichiometric binding (i.e., > 2 × R_max_) were eliminated from further consideration. In total, 95 compounds exhibited ≥ 60% R_max_ (green circles).

To identify compounds with non-specific binding behavior in our SPR screening platform and/or low solubility in aqueous SPR buffers, each compound was initially subjected to a ‘clean screen’. Compounds were first diluted to a final concentration of 500 µM in SPR running buffer. Compounds that were visibly insoluble at this concentration were eliminated from further testing. The remaining compounds were then tested for non-specific binding to a blank flowcell on an SPR sensor chip. Compounds were injected at 500 µM for 30 s and those that exhibited > 5.0 RU residual binding signal at 10 s post injection were considered as non-specific binders and were eliminated from further testing. A total of 381 compounds failed the clean screen (sub-library/number of compounds: 2D-FL/15; 3D-FL/33; NPB/20; SPI/73; PPI/240). The remaining 1619 compounds that passed the clean screen were carried forward to direct C1r-binding assays. Each compound was injected at a concentration of 500 µM over immobilized full-length human C1r. Injections that fell outside of the DMSO solvent correction curve and those that exhibited superstoichiometric binding [36] were eliminated from further consideration. In total, 95 compounds of the original 2000 (4.8%) exhibited ≥ 60% of the theoretical maximal binding response (Figure 2, green circles).

### 2.3. C1r-Binding Properties of Hit Compounds

**Figure 3:**
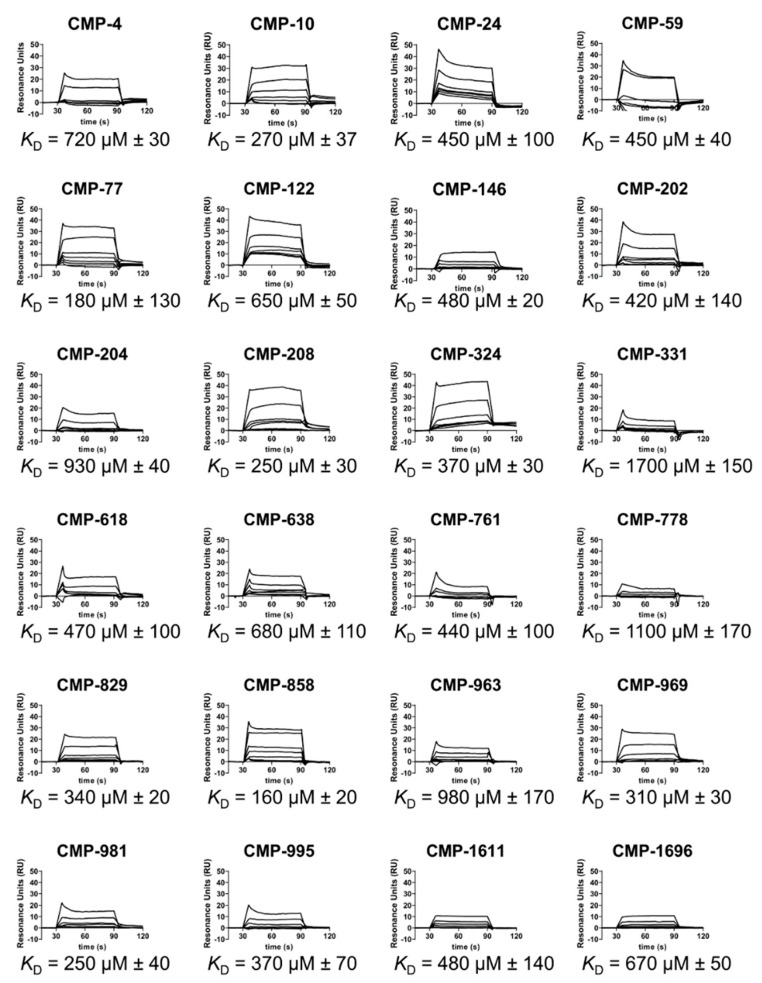
Dose-dependent binding of full-length C1r by selected hit compounds. Dose-dependent C1r binding for 24 compounds was measured by injecting a two-fold variable concentration series of each compound ranging from 7.8 to 500 µM. Steady-state affinities were calculated from the resulting sensorgrams. A representative set of sensorgrams are shown along with the associated steady-state *K*_D_ values. The corresponding steady-state fits are shown in Figure S1. *K*_D_ values are reported as the mean ± S.D. calculated from three independent injection series.

We obtained each of the 95 hit compounds in larger quantities for further characterization. Of these, 24 compounds bound to C1r dose-dependently and fit well to a 1:1 steady-state binding model (Figure 3 and Figure S1). Steady-state affinities (*K*_D_) for these compounds ranged from 160–1700 µM (Figure 3). Clustering analysis revealed that nearly all 24 compounds were structurally distinct from one another (Figure S2), which was expected due to the design criterion of chemical diversity for the fragment library (see above). However, we noted very high similarity between CMP-24 and CMP-202 as judged by an atom pair Tanimoto coefficient of 0.83 [37,38]. Likewise, compounds CMP-761 vs. CMP-981 (Tanimoto coefficient = 0.64) and CMP-618 vs. CMP-638 (Tanimoto coefficient = 0.50) share a high level of similarity.

To better understand where each fragment binds on C1r, we used molecular docking. C1r is a 92 kDa modular serine protease composed of six sequentially arranged domains named complement C1r/C1s, Uegf, Bmp1 (CUB), epidermal growth factor-like (EGF), complement control protein (CCP), and serine protease (SP). While a crystal structure of full-length C1r (i.e., CUB1-EGF-CUB2-CCP1-CCP2-SP) has not been solved, atomic resolution structures of several C1r-domain truncations have been reported, including the N-terminal domains (CUB1-EGF-CUB2; PDB: 6F39) and the remaining C-terminal domains (CCP1-CCP2-SP; PDB: 1GPZ) [39,40,41,42,43].

To limit the conformational search space and to restrict the docking to experimentally derived structures rather than a model of C1r, we carried out two independent in silico experiments. To this end, each of the 24 lead compounds were docked onto the available structures of the N-terminal half of C1r (PDB:6F39) and separately to the C-terminal half of C1r (PDB:1GPZ). In the N-terminal docking experiment, each compound bound to one of two pockets either on the CUB1 domain or at the interface between CUB1-EGF (Figure S3A). In the C-terminal docking experiment, four potential binding pockets were identified across all lead compounds, all of which were found on the C1r-SP domain. Most of the compounds bound to the S1 subsite near the C1r catalytic site (Figure S3B, box 2). In general, the respective N- and C-terminal binding pockets for the top scored poses of each compound were similarly favorable, as judged by the calculated docking binding energies. Notable exceptions included CMP-4, CMP-59, and CMP-981, which have more favorable predicted binding energies of ≤−2.8 kcal/mol for the N-terminal site compared to the corresponding C-terminal site.

### 2.4. Identification of Two Structurally Distinct C1r-Binding and Complement Inhibitory Lead Fragments

**Figure 4:**
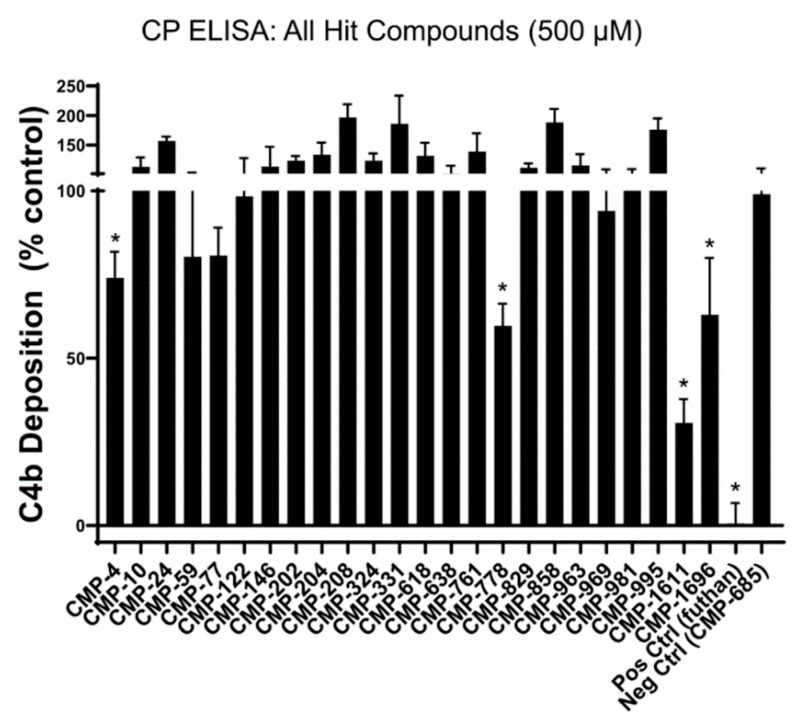
Inhibition of the classical pathway by selected hit compounds. All 24 hit fragments were tested for their ability to block C4 activation in an ELISA-based assay under conditions specific for the classical pathway. Each compound was tested in triplicate at a single concentration of 500 µM. Positive hits (four in total) were defined as any compound that significantly reduced C4b deposition relative to a non-binding control compound (CMP-685), as judged by an unpaired *t*-test (* *p* < 0.05).

**Figure 5:**
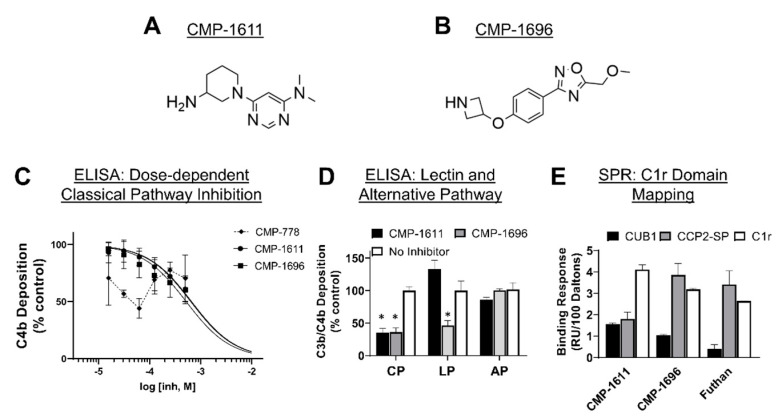
Selectivity and mechanistic analysis of CMP-1611 and CMP-1696. (**A**) Chemical structure of CMP-1611. (**B**) Chemical structure of CMP-1696. (**C**) Dose-dependent inhibition by CMP-1611 and CMP-1696 in a classical pathway-specific ELISA. Data were fit with GraphPad Prism using non-linear regression with a log(inhibitor) vs. response model. For CMP-1611, an IC_50_ value of 660 µM with an associated 95% confidence interval of (560–790 µM, *n* = 9) was calculated. For CMP-1696, an IC_50_ value of 520 µM with an associated 95% confidence interval of (410–680 µM, *n* = 7) was calculated. The CMP-778 inhibitory response could not be fit to a dose–response inhibition model. (**D**) Complement pathway selectivity of CMP-1611 and CMP-1696. Compounds were assessed for their ability to inhibit activation of complement via the lectin and alternative pathways using single doses of 500 µM compound in triplicate. To ensure only lectin pathway activation, 2% (*v*/*v*) C1q-depleted serum (CompTech) was used and mannan was used as the activator. To match serum sources and amounts for this assay, the classical pathway assays were repeated here using serum from CompTech at 2% (*v*/*v*) final concentration. Alternative pathway activation assays were performed using 20% (*v*/*v*) serum (CompTech), alternative pathway buffers, and C3b detection (see Methods and Materials for details). CMP-1611 had no effect on the lectin or alternative pathway, whereas CMP-1696 blocked lectin but not alternative pathway activation. (**E**) C1r, C1r-CUB1, and C1r-CCP2-SP, were immobilized on an SPR sensor chip and binding responses for 500 µM CMP-1611 and CMP-1696 or 10 µM Futhan were each injected in duplicate over all surfaces. Binding responses were corrected for the molecular weight of each analyte and the immobilization level and molecular weight of each surface ligand. Measures of statistical significance in (**D**) were obtained by comparison of vehicle control using an unpaired *t*-test (* *p* < 0.05).

To determine if our lead fragments had direct inhibitory activity against the classical pathway, each compound was tested in a serum-based in vitro assay of complement function using conditions specific for classical pathway activation. Compared to a non-C1r-binding control compound, CMP-4, CMP-778, CMP-1611, and CMP-1696 exhibited statistically significant inhibitory activity when used at a single 500 µM final concentration (Figure 4). We tested the top three most inhibitory compounds (i.e., CMP-788, -1611, and -1696) in a dose–response assay. Two of these compounds, CMP-1611 and CMP-1696, behaved dose-dependently and exhibited half-maximal inhibitory concentrations of 660 and 520 µM, respectively (Figure 5C).

Compounds CMP-1611 and CMP-1696 are structurally distinct from one another (Tanimoto coefficient = 0.09). CMP-1611 is composed of a central pyrimidine group linked to an aminopiperidine moiety, whereas CMP-1696 is composed of a central phenol group linked to an oxadiazole group on one side and an azetidine on the other side (Figure 5 and Figure S2). Each compound is best classified as a small-molecule fragment and each exhibit favorable ‘rule-of-three’ compliant physicochemical properties (i.e., ≤ 300 Da, ≤ 3 hydrogen bond donors, and ≤ 3 clogP) [34].

To assess selectivity of CMP-1611 and CMP-1696, we performed pathway-specific ELISA-based inhibition assays. Neither CMP-1611 nor CMP-1696 inhibited complement under conditions that selectively activate the alternative pathway, suggesting a level of specificity for the classical pathway that would be expected based on the C1r-binding properties of each compound (Figure 5D). However, CMP-1696, but not CMP-1611, blocked lectin pathway activation at a similar level as it did the classical pathway (Figure 5D). To further investigate the divergent selectivity profiles of CMP-1611 and CMP-1696, we first sought to clarify the binding site on C1r for each compound. Both compounds were predicted to bind the same pocket on the CUB1 domain in the N-terminal C1r molecular docking experiment. Likewise, the C-terminal docking experiment predicts that CMP-1611 and CMP-1696 bind within the S1 subsite of the C1r-SP domain. To test these predictions, we produced recombinant C1r-domain truncations that included the predicted N-terminal binding site (C1r-CUB1 domain) or the C-terminal site (C1r-CCP2-SP). On the same biosensor chip, we immobilized C1r-CUB1, C1r-CCP2-SP, and full-length C1r on three different flow cells. We then injected each compound simultaneously over the three surfaces at 500 µM and measured binding responses. As a control, we also injected 10 µM Futhan (FUT-175), which is a promiscuous serine protease inhibitor previously reported to inhibit C1r and which is proposed to bind the active site of its serine protease targets (Figure S1B) [44]. Indeed, comparison of the relative binding responses showed that Futhan bound C1r and C1r-CCP2-SP similarly, whereas very little binding response was seen for the C1r-CUB1 domain (Figure 5C). The domain mapping profile for CMP-1696 was similar to that of Futhan, with CMP-1696 exhibiting high binding to C1r and C1r-CCP2-SP but low binding to CUB1 (Figure 5E). In contrast, CMP-1611 bound weakly to both C1r-CUB1 and C1r-CCP-2SP relative to full-length C1r (Figure 5E).

### 2.5. C1r-Binding Mode of CMP-1696

**Figure 6:**
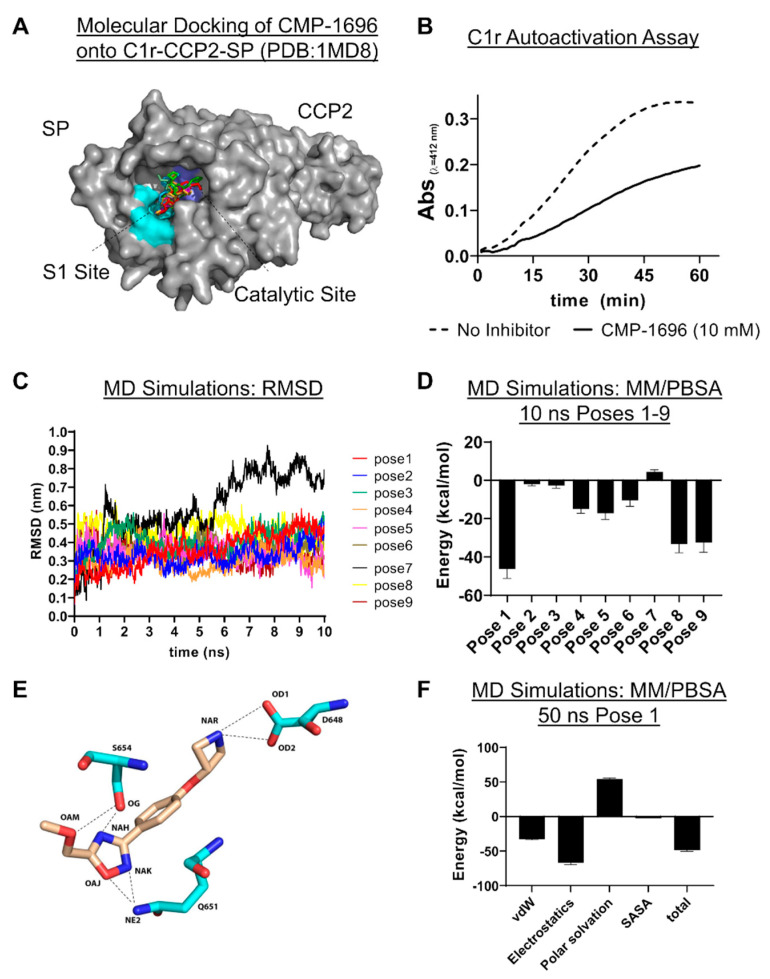
CMP-1696 structure activity relationship. (**A**) CMP-1696 was redocked onto C1r-CCP2-SP (PDB: 1MD8, grey surface representation) and the top nine scored poses are shown. All CMP-1696 poses dock into the S1 subsite (cyan) near the catalytic triad (blue). (**B**) C1r autoactivation assay. C1r proenzyme undergoes time-dependent autoactivation at 37 °C. Autoactivation was measured using a synthetic substrate for C1r enzyme. The reaction progress of vehicle control (dashed line) or in the presence of 10 mM CMP-1696 (solid line) was monitored for 1 h. (**C**) Molecular dynamics (MD) simulations of CMP-1696/C1r-CCP2-SP. Root mean square deviation (RMSD) in nm for each of the CMP-1696 poses measured over the 10 ns molecular dynamics simulation. (**D**) MM/PBSA energy calculations for each pose in the 10 ns MD simulations indicate that pose 1 is the most energetically favorable. (**E**) Hydrogen bonding interactions at the start of the MD simulation are shown as dashed lines. (**F**) A 50 ns MD simulation (Video S1) was carried out for pose 1 and MM/PBSA was used to calculate total energy. Subcategorized energy contributions are also shown where vDW is van der Waals forces and SASA is solvent-accessible surface area.

Collectively, the data above showed that CMP-1611 is selective for classical pathway inhibition but that its interaction site on C1r and mechanism of action remain ambiguous. By contrast, an analogous set of experiments for CMP-1696 is consistent with this compound binding to the S1 subsite on C1r and potentially to serine proteases of the lectin pathway. In an effort to support future development of CMP-1696 based compounds into larger drug-like molecules that selectively inhibit C1r, we carried out a series of molecular docking and molecular dynamics (MD) simulations. The experimental data obtained above showed that CMP-1696 binds directly to C1r-CCP2-SP (Figure 5E), indicating that the CCP1 domain does not contribute to CMP-1696 affinity. Thus, to minimize computational resources in our MD simulation experiments we redocked CMP-1696 onto the available crystal structure of the CCP2-SP region of C1r (PDB:1MD8) (Figure 6A). The top nine poses for CMP-1696 bound within the S1 pocket and have similar calculated docking energies. Consistent with an S1 binding site on C1r, 10 mM CMP-1696 directly inhibited the autocatalysis of C1r proenzyme (Figure 6B).

During an MD simulation, the system comprising the ligand (i.e., CMP-1696) and the protein (i.e., C1r-CCP2-SP), is set into motion with initial velocity. The trajectory and velocity of the system are then tracked, allowing for characterization of dynamic interactions between the ligand and the protein. Identification of the correct binding pose resulting from a molecular docking experiment may be improved by rescoring the ensemble of poses using a combination of MD simulations and molecular mechanics Poisson–Boltzmann/Surface Area (MM/PBSA) free binding energy calculations [45,46]. To further investigate the true CMP-1696 binding mode, we carried out short 10 ns MD simulations for each of the top nine scored docking poses (Figure 6C,D) and used MM/PBSA to calculate protein–ligand binding energies. Aside from pose 7, all CMP-1696 docking poses are predicted to be energetically favorable with pose 1, ultimately agreeing with the docking scoring as the most favorable (Figure 6D). This binding pose, shown in Figure 6E, forms a total of six hydrogen bonds including two between the CMP-1696 azetidine group and sidechain atoms of D648. The D648 residue is functionally important as it forms the bottom of the S1 pocket in C1r [42]. The CMP-1696 oxadiazole group forms several hydrogen bonds including with the catalytic serine (S654) as well as with Q651(Figure 6E). The interaction with Q651 is notable as this residue is not conserved in either C1s or MASP-2 (Figure S4A). To explore these differences further, we docked CMP-1696 onto the crystal structures of C1s-CCP2-SP and MASP-2-CCP2-SP (Figure S4B–D). Analysis of the top scored binding pose predicts that CMP-1696 binds MASP-2 in a nearly identical conformation as to that observed in C1r-CCP2-SP involving six homologous contact residues (four identical). In contrast, the docking of CMP-1696 onto C1s indicates an alternative binding mode near, but not in, the S1 subsite and with only a single contact residue (K629) in a homologous position to C1r.

To further analyze the binding mode of CMP-1696, we carried out a longer 50 ns simulation using docking pose 1 (Videos S1 and S2). MM/PBSA analysis of this simulation further highlighted the importance of the polar interactions mentioned above in the CMP-1696/C1r interaction (Figure 6F). A more detailed analysis of the simulation reveals a number of interesting features that may help guide the development of CMP-1696 for improved C1r affinity and specificity. An analysis of each hydrogen bonding interaction shown in Figure 6E was conducted across the entire simulation (Figure S6). This revealed that while both hydrogen bonds mediated by D648 are maintained throughout the course of the MD simulation, the hydrogen bonds mediated by Q651 and S654 are predicted to be relatively unstable (Figure S6). From the ligand side of the interaction, we noted that all atoms of CMP-1696 interact with one or more protein atoms in ≥ 50% of the simulation with the notable exceptions of the oxygen (atom label: OAM) and terminal carbon (atom label: CAN) atoms on the 5-methoxymethyl substituent of the oxadiazole group. These atoms form transient contacts across 14 and 21 C1r residues, respectively. Moreover, several transient interactions were identified near this moiety that were not present in the original docking pose and include Ile-483, His-484, Gly-485, Gly-487, Ala-500, Pro-506, Glu-508, and His-509. In contrast, the interaction of the nitrogen atom (atom label: NAR) forms an extremely stable interaction with the ASP-648 (97% of the simulation) and stable interactions with Ala-649 (84%) and Gly-679 (53%), while forming only one transient interaction (Tyr-684, 4%). Collectively, the results of the MD simulation suggest that modification of the oxadiazole ring may be a promising fragment growth strategy.

## 3. Discussion

The field of complement-directed therapeutics has grown considerably over the past decade, which is exemplified by latest estimates of over three dozen complement-targeted drugs in various stages of clinical development [7,8,17,18,47]. This growth has been sparked by an increased understanding of the relationship between complement and human diseases, along with the remarkable clinical successes of eculizumab/Soliris. Despite the burgeoning pipeline, drugs targeting the activation pathways are underrepresented, with a vast majority of current drug discovery efforts being devoted to targeting complement at the level of C3 or C5 [17,47]. Complement inhibitors that halt the cascade at the most upstream initiation steps may be ideal therapies for pathway specific-mediated complement conditions and impart the added advantage of leaving other activation pathways available for complement’s critical role as a sentinel against pathogens.

The classical pathway of complement has been implicated as a driver of several human diseases [12,16,21,22,23,24,25]. Development of drugs that specifically target classical pathway components is currently limited to recombinant and native preparations of C1-INH (Cinryze/Berinert/Ruconest), an anti-C1q antibody ANX005 (Annexon Biosciences, San Francisco, CA, USA) and an anti-C1s antibody TNT009/BIVV009 (True North Therapeutics, San Francisco, CA, USA). C1-INH replacement therapy is FDA approved for the treatment of hereditary angioedema, a disorder not primarily driven by complement; however, evaluation of off-label uses for C1-INH in complement-mediated diseases are ongoing [7,47]. ANX005/ANX007 is being investigated in complement-mediated neurodegenerative disorders, while TNT009/BIVV009 has reached phase 3 clinical trials for cold agglutin disease and is being evaluated in other antibody-driven complement-related conditions, such as bullous pemphigoid [7,47]. In sum, the current landscape of classical pathway-specific drug development is characterized by a limited but promising class of large proteins/antibodies in early clinical development for treatment of an increasing number of complement-related conditions.

The mechanisms underlying the role of complement in human diseases are often complex, disease-specific, and in many cases, poorly understood. In some classical pathway-related diseases, autoantibodies activate complement via C1q/immune complex binding causing attack of healthy host tissues. In other cases, the involvement of the classical pathway may arise from C1q binding to disease-associated non-antibody ligands capable of activating C1 and initiating downstream complement activation. While the long-term goal of developing therapeutics is paramount, successful identification of high-affinity small-molecule inhibitors specific for C1r would allow complement researchers to better understand the role of the classical pathway initiator protease in numerous models of disease.

In this study, we set out to expand the classical pathway-specific therapeutic toolkit by targeting C1 activation at the level of C1r with small-molecule inhibitors. As the initiating protease of the classical pathway, C1r catalyzes the first proteolytic cleavage event and thus represents the most upstream enzymatic target of the pathway. While complement-directed therapeutics continue to be dominated by antibody-based drugs, small-molecules have recently seen two major breakthroughs in the successful development of factor B and factor D-specific inhibitors [48,49,50]. In general, small-molecule drugs are afforded greater tissue penetrance relative to antibodies/biologics and can more easily cross the blood–brain barrier [51,52]. In certain complement-related disease settings, such as neurological disorders, these are likely critical considerations. Additionally, small molecules are more easily formulated into oral medications, which could increase patient compliance and lower costs associated with these therapies. However, there are currently no small-molecule inhibitors that specifically target the classical pathway proteases.

To begin to address this gap, we carried out an unbiased screen to identify novel small-molecule fragments that bound directly to C1r. We chose a FBDD approach, as fragment hits are often better positioned than larger, more chemically complex hits, to be optimized with both affinity and specificity considerations in mind. Additionally, we sought to potentially exploit the fact that C1r’s function as the initiating protease of the classical pathway is constrained by its unique orientation within the C1 complex, which could allow for the discovery of allosteric small-molecule inhibitors. Our initial SPR-based FBDD screen resulted in the identification of 95 compounds with C1r-binding capacity. Ultimately, 24 compounds dose-dependently bound full-length C1r with affinities ranging between 160–1700 µM and ligand efficiencies between 0.16 and 0.41 (Figure S2) [53]. Two fragments emerged as leads and exhibited similar C1r affinities (*K*_D_, CMP-1611 = 480 and *K*_D_, CMP-1696 = 670 µM) and potencies (IC_50_, CMP-1611 = 660 µM and IC_50_, CMP-1696 = 520 µM) (Figure 2, Figure 3 and Figure 5C). CMP-1611 was selective for the classical pathway; however, both its binding and inhibitory modes are currently unclear. In contrast, the data for CMP-1696 suggests that this fragment binds within the S1 pocket of C1r and directly blocks C1r autoactivation (Figure 6). Although CMP-1696 also blocks the lectin pathway, our molecular docking and molecular dynamics studies have provided insight into fragment optimization strategies for C1r-specific compounds.

While the compounds identified here represent high quality fragment hits, we note that several limitations remain for the continued development of these leads. The first challenge is common to nearly all FBDD projects in the need to optimize affinity and potency. In this regard, all 24 C1r-binding fragments identified here benefit from having favorable physiochemical properties (Table S1), making them generally well-suited for further hit-to-lead development. Moreover, while directly growing an individual fragment by adding substituent groups is one strategy for fragment optimization, it is also possible to link or merge separate hits [54]. Thus, the identification here of structurally distinct compounds, many of which are predicted to bind C1r within close proximity to one another, strongly supports this possibility. Another key challenge to overcome will be that of specificity. Due to the conserved nature of the catalytic domains of serine proteases, compounds like CMP-1696 that target the specificity pockets or catalytic sites, increase the possibility of off-target effects. Indeed, CMP-1696 inhibits the lectin pathway and is predicted to adopt a nearly identical binding mode on MASP-2 (Figure 5D and Figure S4D). However, given their low molecular complexity, it is not necessarily expected that fragment hits themselves will exhibit high selectivity [36]. Nonetheless, it is encouraging that active-site targeted small-molecule inhibitors of other complement serine proteases, factor B and factor D, have ultimately overcome this same challenge of specificity [48,49,50]. Although our C1r-domain mapping and molecular dynamics studies have provided information about CMP-1696, defining the binding mode of each fragment hit identified here—including CMP-1611 using empirical experimental methods, such as x-ray crystallography coupled with rigorous MD simulations—is a key next step being actively pursued in our laboratory.

In summary, we report the identification of 24 novel small-molecule fragments that bind directly to the initiator protease of the classical pathway of complement, C1r. Two of these fragments, termed CMP-1611 and CMP-1696, directly inhibit the classical pathway but exhibit different selectivity and may act by different mechanisms. Each of the 24 hit compounds are small (161–406 Da) and have favorable physicochemical properties, and therefore have strong potential to be optimized independently or in combination. The developmental evolution of one or more of these compounds may ultimately yield valuable research tools and potentially novel treatment options for diseases associated with aberrant classical pathway activation.

## 4. Materials and Methods

### 4.1. Recombinant Expression, Purification, and Refolding of C1r-Domain Truncations

Purified C1r and C1r proenzyme were purchased from Complement Technology (CompTech). Recombinant C1r-domain truncations were produced by sub-cloning an *Escherichia coli* codon-optimized synthetic oligonucleotide (IDT Technologies, Coralville, IA, USA) flanked with a 5′ BamHI site, a 3′ NotI site and a stop codon into the pT7HMT vector [55]. The C1r-CUB1 construct corresponds to C1r residues 18–141, whereas the C1r-CCP2-SP construct corresponds to C1r residues 307–705 (UNIPROT numbering: no. P00736). C1r-domain truncations were purified under denaturing conditions using previously published protocols with some modifications [39,56]. Plasmids were transformed into *E. coli* BL21 (DE3) and cells were grown to an optical density of 0.6–0.8 OD600 at 37 °C in Terrific Broth supplemented with kanamycin. Cultures were then induced overnight with isopropyl β-D-thiogalactoside and cells were collected by centrifugation for 10 min at 4000× *g*. Supernatants were discarded and resuspended in 100 mL of lysis buffer (6 M guanidine HCl, 100 mM Tris pH 8.0, 10 mM imidazole) for 30 min and clarified by centrifugation for 30 min at 16,000× *g*. Nickel NTA beads (GoldBio, St. Louis, MO, USA) (5 mL column volume) were washed with 25 mL of denaturing binding buffer (8 M urea, 500 mM NaCl, 20 mM sodium phosphate pH 6.0, 10 mM imidazole). Samples were then passed over columns at a flow rate of 1–2 drops per second and afterwards washed with another 25 mL of denaturing binding buffer. Samples were eluted with 5 mL of denaturing elution buffer (8 M urea, 500 mM sodium chloride, 20 mM sodium phosphate pH 6.0, 200 mM imidazole) and then rapidly diluted 1:10 into refold buffer containing 50 mM Tris pH 8.3, 3 mM reduced glutathione, 1 mM oxidized glutathione, 5 mM ethylenediaminetetraacetic acid (EDTA), and 500 mM arginine. The next day, samples were dialyzed twice for four hours at 25 °C against 2 L of 50 mM Tris-HCl pH 7.4, 145 mM NaCl, and concentrated to less than 12 mL using 10 kDa molecular weight cutoff ultracentrifugation filters (Millipore). Refolded protein was then purified using an ÄKTA pure Fast Pressure Liquid Chromatograph (FPLC, GE Healthcare, Chicago, IL, USA) connected to a HiLoad 26/600 Superdex 75 pg column previously equilibrated in 200 mM sodium chloride and 20 mM Tris pH 8.0. For purification of C1r-CUB1, 5 mM CaCl_2_ was added to all native buffers. Fractions were evaluated by SDS-PAGE and those containing the C1r truncation mutant were pooled. To remove affinity tags, tobacco etch virus (TEV) enzyme (previously activated with 1 mM DTT) was incubated with pooled refolded protein overnight at 25 °C. FPLC nickel affinity chromatography was carried out the following day and the unbound fraction was collected, concentrated by ultracentrifugation, buffer exchanged into 20 mM HEPES (pH 7.3), 140 mM NaCl, aliquoted, and stored at −80 °C until use. Sequence alignments were performed with EMBL-EBI Clustal Omega [57].

### 4.2. Compound Library

Small-molecule compounds were obtained from ChemDiv Inc. All compounds were received in pre-weighed vials as powder, dissolved in dimethyl sulfoxide (DMSO) (GoldBio, Louis, ST, USA) to a final concentration of 10 mM and stored at –20 °C. Representations of compounds were prepared using ChemDraw Prime 19.1 (Perkin Elmer, Waltham, MA, USA). Structural comparisons of compounds were carried out with the ChemMine software suite [58]. Hierarchical clustering using a single linkage was used to generate a distance matrix using Cluster, and atom pair Tanimoto coefficients [37,38] were calculated using the Similarity Workbench. Physicochemical descriptors of each compound in the library were analyzed using SwissADME (Table S1) [59]. Pan Assay Interference analysis was performed by SwissADME and is reported in Table S1 [59].

### 4.3. Surface Plasmon Resonance

All SPR experiments were carried out using a Biacore T200 (GE Healthcare, Chicago, IL, USA) at 25 °C. For all experiments, a running buffer (HBS-T) of 20 mM HEPES (pH 7.3), 140 mM NaCl, 0.005% (*v*/*v*) Tween-20, 5% DMSO (*v*/*v*) was used. For each experiment, DMSO calibration curves ranging from 4% to 5.5% DMSO (*v*/*v*) were collected at the beginning, end, and every 50 cycles throughout the duration of the experiment. All experiments were performed using HC1500M sensor chips (Xantec, Duesseldorf, Germany). In total, 10 sensor chips and 16 ligand immobilized surfaces were created over the course of this study (detailed in Table S2). In all cases, C1r proteins were immobilized onto chip surfaces using amine-coupling chemistry with 1-ethyl-3-(3-dimethylaminopropyl)-carbodiimide) (EDC), N-hydroxysuccinimide (NHS), followed by ethanolamine. Briefly, 100 mM of EDC and NHS were mixed and injected to activate the chip surface followed by the C1r protein ligand captured at a defined resonance unit (RU) level, followed by reaction quenching with 100 mM ethanolamine pH 8.5. Flow cell 1 was always used as a reference cell (no ligand), unless otherwise noted. All sensorgrams were reference and blank subtracted and analyzed using T200 Evaluation Software (GE Healthcare, Chicago, IL, USA).

### 4.4. Initial SPR Screening

Using 96-well plates, 5 µL of each of the 2000 fragment compounds were diluted with 95 µL of 1.05 × HBS-T. This provided individual 500 µM solutions of each compound with a final DMSO concentration of 5% (*v*/*v*) to match the running buffer. All compounds were first visually inspected for solubility in running buffer, and insoluble compounds were not included in the subsequent ‘clean screen’. To determine if compounds nonspecifically interacted with the chip surface, a ‘clean screen’ was performed using a single flowcell on an uncoupled HC1500M. Compounds which exhibited > 5.0 RU of residual binding at 10 s post injection were removed from the subsequent screen. Approximately 1600 compounds passed this selection criteria. These compounds were then screened at 500 µM for binding to full-length C1r enzyme immobilized at high density (see Table S2). Baseline noise was accounted for using T200 evaluation software. A theoretical maximal binding signal (R_max_) was calculated using the equation R_max_ = (C1r immobilization level (RU) × (mol. wt. compound/mol. wt. C1r) * *n*), where the C1r immobilization is reported in Table S2 for each surface used, mol. wt. C1r = 92,000 Da, and *n* is the binding stoichiometry, assumed here to be 1. Compounds were considered as hits if all the following criteria were met: i) injections fell within the DMSO calibration curve; ii) did not exhibit > 5.0 RU of residual binding to the reference surface; iii) did not exhibit abnormal sensorgram shape; and iv) did not exhibit superstoichiometric binding [60]. 

### 4.5. Evaluation of Dose-Dependent Binding by SPR

To further evaluate hit compounds from the initial SPR screen, dose-dependent SPR binding assays were carried out. Three replicate flow cells were used containing both low and high immobilization densities of C1r (see Table S2). Hit compounds were tested for dose-dependent binding to full-length C1r using a compound concentration range of 7.8–500 µM. T200 evaluation software was used to calculate steady-state affinities for each compound by fitting sensorgrams from each variable concentration injection dataset using a 1:1 Langmuir model of interaction constrained by a theoretical experimental binding response based on the mol. wt. of each compound and derived as stated above. *K*_D_ values are reported as the mean ± the standard deviation from at least three independent measurements.

### 4.6. Complement Inhibition Assay

To carry out classical pathway ELISA-based inhibition assays, IgM was dissolved in coating buffer (100 mM Na_2_CO_3_/NaHCO_3_, pH 9.6) to a final concentration of 3 µg/mL and was dispensed into 96-well plates at 100 µL per well. Mannan was dissolved to 2 µg/mL and lipopolysaccharides (LPS) were dissolved to 10 µg/mL in coating buffer for use in lectin and alternative pathway inhibition assays, respectively. Immobilization was allowed to proceed overnight at 25 °C. The immobilization reagent was then removed, followed by three washes in washing buffer (50 mM Tris pH 8.0, 200 mM NaCl, 0.05% (*v*/*v*) Tween-20), and blocked using 100 µL of 1% (*w/v*) bovine serum albumin in PBS-T for 1 h at 37°C. Blocking buffer was removed and plates were washed three times in washing buffer. Next, 500 µM of each compound, 5% (*v*/*v*) DMSO, and 1% (*v*/*v*) normal human serum (Innovative Research) (final concentrations) were dissolved in classical/lectin pathway ELISA buffer (20 mM HEPES (pH 7.3), 0.1% (*w/v*) gelatin, 140 mM NaCl, 2 mM CaCl_2_, 0.5 mM MgCl_2_) and added to the IgM-coated plate for 1 h at 37 °C. For the lectin pathway-specific ELISAs shown in Figure 5D, we used C1q-depleted serum from CompTech at 2% (*v*/*v*) and thus the classical pathway assays for this analysis were also performed using serum from CompTech at 2% (*v*/*v*). For the alternative pathway, 20% (*v*/*v*) normal human serum (CompTech) (final concentration) and alternative pathway ELISA buffer (20 mM HEPES, (pH 7.5), 0.1% (*w/v*) gelatin, 140 mM NaCl, 2 mM CaCl_2_, 0.5 mM MgCl_2_) were used in place of classical/lectin pathway ELISA buffer. Plates were then washed three times in wash buffer and each well was filled with 100 µL of α-C4 (HYB 162-02, Santa Cruz Biotechnology) diluted 1:300 for classical and lectin pathway assays and 100 µL of C3b (WM-1, Sigma) diluted 1:300 for alternative pathway assays. Plates were again incubated at 37 °C for 1 h, washed three times, filled with 100 µL of HRP-conjugated goat-α-mouse secondary antibody (diluted 1:3000) (Thermo Scientific, Waltham, MA, USA), and rocked gently for 1 h at 25 °C. After three more washes, 50 µl of substrate (1-step Ultra TMB, Thermo Scientific, Waltham, MA, USA) was added to each well and rocked for 15 min in the dark at 25 °C. The reaction was stopped with 50 µL of 0.16 M sulfuric acid, and the plate was read at 450 nm using an EnSight multimode plate reader (Perkin-Elmer). Each column contained a positive control for full complement activity (5% (*v*/*v*) blank DMSO with 1% (*v*/*v*) serum) and a negative control for no complement activity (5% (*v*/*v*) blank DMSO and no serum). Positive controls (1% (*v*/*v*) serum, no inhibitor) were defined as 100% C4b or C3b signal whereas negative controls (no serum) were defined as 0% C4b or C3b signal for each column. Compounds that inhibited complement deposition relative to the control were then evaluated using the same assay setup across a two-fold variable concentration series for each compound (7.8–500 µM). These data were used to obtain a half maximal inhibitory concentration (IC_50_) by fitting dose–response curves using inhibitor vs. response models in GraphPad Prism 8. All experiments were performed no less than three times.

### 4.7. Molecular Docking

Compound structures in 2D SDF formatted images were converted to 3D MOL2 formatted files using OpenBabel v 2.4.1 [61]. MOL2 formatted files were then converted to PDBQT file format using the prepare_ligand.py script from AutoDockTools 1.5.6 [62]. Protein structure files for the N-terminal portion of C1r (CUB1-EGF-CUB2; PDB: 6F39) and the C-terminal region of C1r (CCP1-CCP2-SP; PDB: 1GPZ) were prepared for docking by removing water molecules and adding hydrogens using AutoDockTools 1.5.6 before generating protein structure PDBQT files. Binding boxes for molecular docking were designed to encompass the entirety of the protein structures. Coordinates for the center (x, y, z) and size (x, y, z in Å) of target boxes are; N-terminal region of C1r: center (135, 100, 40), size (82, 62, 92), and C-terminal region of C1r: (55, 10, 33), size (98, 70, 66). Molecular docking was then performed using AutoDock Vina 1.1.2 [62] and as detailed previously [63]. Nine poses for each compound were collected and included in the full dataset. Molecular docking of CMP-1696 onto the C1r-CCP2-SP crystal structure was performed using PDB:1MD8. A grid box encompassing only the SP domains of the receptor were chosen with center (26, −2, 12) and a box length of (40, 44, 38). The grid size spacing was set to 1 Å, with exhaustiveness set to 8 and an energy range of 4 kcal/mol. Molecular docking was then performed with the Vina executable embedded with PyRx v0.9.8 [64] resulting in nine binding poses for each compound. All representations of protein structures were prepared using PyMOL Molecular Graphics System, Version 2.0 Schrödinger, LLC (www.pymol.org/).

### 4.8. C1r Proenzyme Activation Assay

Individual stock solutions of 1 mM of Z-Gly-Arg-sBzl (MP Biomedicals, Irvine, CA, USA) and 5,5′-Dithiobis-(2-nitrobenzoic acid) (DTNB) (Sigma-Aldrich, St. Louis, MO, USA) were made by dissolving powder in DMSO to give 10 mM stocks of each compound, which were then diluted to 1 mM in assay buffer (20 mM HEPES pH 7.3, 140 mM NaCl, 0.005% (*v*/*v*) Tween-20, 2 mM CaCl_2_). In 96-well plates, 5 µL of 100 mM compound stocks (in DMSO) were added into wells in triplicate along with 25 µL of 50 nM proenzyme C1r (stored over ice until use). To these wells, 5 µL of 1 mM DTNB and 5 µL of 1 mM Z-Gly-Arg-sBzl were added simultaneously. Another 10 µL of assay buffer was added to each well to give a total reaction volume of 50 µL. Reactions were carried out at 37 °C. Plates were read at 450 nm using an EnSight multimode plate reader (Perkin-Elmer, Waltham, MA, USA) and the reaction progress was tracked for 1 h.

### 4.9. Molecular Dynamics 

Molecular dynamics simulation of C1r-CCP2-SP/CMP-1696 binding were performed with Gromacs 2019.3 [65]. For C1r simulation, the high-resolution crystal structure with PDB code:1MD8 was used and missing residues were built using Coot v0.8.9.2 [66]. Simulations of the nine top poses from the CMP-1696 docking onto C1r-CCP2-SP were performed at 300 K for a duration of 10 ns, while pose 1 was eventually carried out to 50 ns. The topology files for the receptors were prepared with Gromos 43a1 united atom force field, while that of compound CMP-1696 was built using the PRODRG server [67]. The protein–ligand complex system was then solvated in a dodecahedron box with dimensions of 49.9 Å, 46.6 Å, and 76.4 Å with TIP3P model waters (26,734 water molecules) using periodic boundary conditions of 10 Å from the edge of the solvation box. The system was charge neutralized by replacing eight Na^+^ ions in place of TIP3P water molecules. Bond lengths were constrained by the LINCS algorithm [68] and all long-range electrostatics were determined using the smooth particle mesh Ewald (PME) method [69]. Energy minimization was performed with the steepest descent algorithm until convergence (~1000 steps). Temperature equilibration was conducted by the isochoric-isothermal NVT ensemble (constant number of particles, volume, and temperature) with a Berendsen thermostat [70] for 100 ps. The system was then subjected to pressure equilibration in the NPT ensemble (constant number of particles, pressure, and temperature) for 100 ps using the Parrinello–Rahman protocol [71], maintaining a pressure of 1 bar. The top-pose of CMP-1696, which also turned out to yield the highest binding energy through MM/PBSA [72] energy calculations, was chosen for a production MD run of 50 ns with snapshots being saved at 2 ps intervals. Backbone RMSD, intra-protein hydrogen bonds and trajectory analyses were performed with GROMACS programs ‘gmx rms’, ‘gmx hbond’, and ‘gmx trjconv’. Hydrogen bonds between atoms of CMP-1696 and C1r-CCP2-SP were calculated using “gmx distance” program in Gromacs 2019.3.

Binding energy calculations for the receptor-compound complex were carried out with g_mmpbsa program [72]. Initially, for all the docked poses, the entire simulation trajectories were subjected to these calculations with an interval of 100 ps, amounting to 100 snapshots. For the 50 ns MD simulation, trajectories were sampled at 100 ps, amounting to 500 snapshots. All components of binding energy categorized into non-polar, polar, and solvent-accessible surface area were calculated using standard protocols employed by the g_mmpbsa program. Error estimates for MM/PBSA calculations were performed via bootstrapping methods for 2000 steps according to guidelines outlined in g_mmpbsa program. All subsequent data analyses were performed using in-house codes written in Fortran 90, Python and C-shell scripts.

### 4.10. Statistics

Statistical analyses were performed using GraphPad Prism version 8. For complement inhibition assays, measures of statistical significance for single-dose experiments were assessed using unpaired Student’s *t*-tests compared to no inhibitor controls (pathway ELISAs) or non-binding control compounds (classical pathway ELISA). Statistical significance was defined as *p* < 0.05. Calculation of IC_50_ values from dose-dependent complement assays were obtained by non-linear regression analysis using a using an inhibitor vs. response model and with 95% confidence intervals reported. For non-linear regression analyses, the top and bottom of each curve were constrained to 100 and 0, respectively.

## Supplementary Materials

The following are available online; Figure S1: Steady-state affinity fits for compounds evaluated for C1r dose-dependent binding; Figure S2: Structures of C1r-binding hit fragments identified in this study; Figure S3: Molecular docking studies of compounds with C1r-CUB1-EGF-CUB2 and C1r-CCP1-CCP2-SP; Figure S4: Sequence alignment of the serine protease domains of C1r, C1s, and MASP-2; Figure S5: MD simulation for CMP-1696/C1r-CCP2-SP; Figure S6: Analysis of hydrogen bonding interactions during the CMP-1696/C1r-CCP2-SP MD simulation; Table S1: SwissADME analysis of the 2000 compound library; Table S2: SPR Sensor chips used in this study; Video S1: MD Simulation of CMP-1696/C1r-CCP2-SP for 50 ns; Video S2: Docking of CMP-1696 onto C1r-CCP2-SP (pose1).
