# Supplementary material for: Targeting the Initiator Protease of the Classical Pathway of Complement Using Fragment-Based Drug Discovery
Source: Molecules. 2020 Sep 3;25(17):4016. doi: 10.3390/molecules25174016 (PMC7504721; doi:10.3390/molecules25174016)
Supplement: Supplementary file 1 [file molecules-25-04016-s001.zip › Rushing and Rohlik et. al - C1r FBDD Supplemental Figures- FINAL_revisions.pdf]

Supplementary Figure Legends

Supplementary Figure 1. Steady-state affinity fits for compounds evaluated for C1r dose-dependent binding. (A) Representative steady-state affinity fits that correspond to the sensorgrams shown in Fig. 3. Affinity fits were calculated using T200 Evaluation Software assuming 1:1 binding interactions and the molecular weight corrected theoretical maximal binding response of each analyte. (B) A representative set of sensorgrams for dose-dependent binding of full-length C1r by futhan/FUT-175 and associated steady-state affinity fit. K_D values are reported as the mean ± S.D. calculated from three independent injection series.

Supplementary Figure 2. Structures of C1r-binding hit fragments identified in this study. Structures of each of the 24 dose-dependent C1r-binding compounds identified in this study. Ligand efficiency (LE) = (ΔG/number of non-hydrogen atoms), where ΔG = -RT x ln $K_{D,C1r,}$ using K_D values reported in Fig. 3. All structures were drawn using ChemDraw Prime 19.1.

Supplementary Figure 3. Molecular docking studies of compounds with C1r-CUB1-EGF-CUB2 and C1r-CCP1-CCP2-SP. Each of the 24 C1r-binding compounds was mapped onto either the (A) N-terminal region of C1r (CUB1-EGF-CUB2 (PDB: 6F39)) or (B) C1r (CCP1-CCP2-SP) (PDB: 1GPZ)). The lowest-energy binding pose for each compound is shown in each of the respective *in silico* experiments. All compounds bound to one of two sites on the N-terminal region of C1r or one of four SP domain sites in the C-terminal docking. Binding locations are outlined by red dashed boxes.

Supplementary Figure 4. Sequence alignment of the serine protease domains of C1r, C1s, and MASP-2. (A) Residues that participate in sidechain interactions with pose 1 of CMP-1696/C1r-CCP2-SP are highlighted with blue boxes and red backgrounds. These include H502, T503, D648, Q651, and S654. Among these Q651 and T503 are unique to C1r. Clustal Omega was used to generate the alignment. (B) Molecular docking of CMP-1696 onto C1r (PDB:1MD8) (C) Molecular docking of CMP-1696 onto C1s (PDB: 1ELV) or (D) MASP-2 (PDB:1Q3X). Residues within 4.0 Å of CMP-1696 are shown where homologous residues positions in C1s and MASP-2 relative to C1r are marked in red (identical residues) or green (non-identical residues).

Supplementary Figure 5. MD simulation for CMP-1696/C1r-CCP2-SP (A) Snapshots from the 50 ns MD simulation for CMP-1696(pose1)/C1r-CCP2-SP are shown in 10 ns intervals. See Supplemental Movie 1 for full simulation. (B) RMSD of the backbone ligand (nm) for duration of the 50 ns MD simulation. (C) Root mean square fluctuations (RMSF) in nm shown for C1r residues using C1r numbering from the 1MD8 PDB file.

Supplementary Figure 6. Analysis of hydrogen bonding interactions during the CMP-1696/C1r-CCP2-SP MD Simulation. A-F) Hydrogen bonds between CMP-1696 and C1r-CCP-2SP monitored over the duration of the 50 ns simulation. Hydrogen bond donor-acceptors were identified within a distance cutoff of 0.35 nm, these distances were then traced with "gmx distance". The average distance of each hydrogen bond donor-acceptor pair is reported ± S.D.

Supplementary Video 1. MD Simulation of CMP-1696/C1r-CCP2-SP for 50 ns.

Supplementary Video 2. CMP-1696 pose 1 docked onto 1MD8.

Supplementary Fig. 1

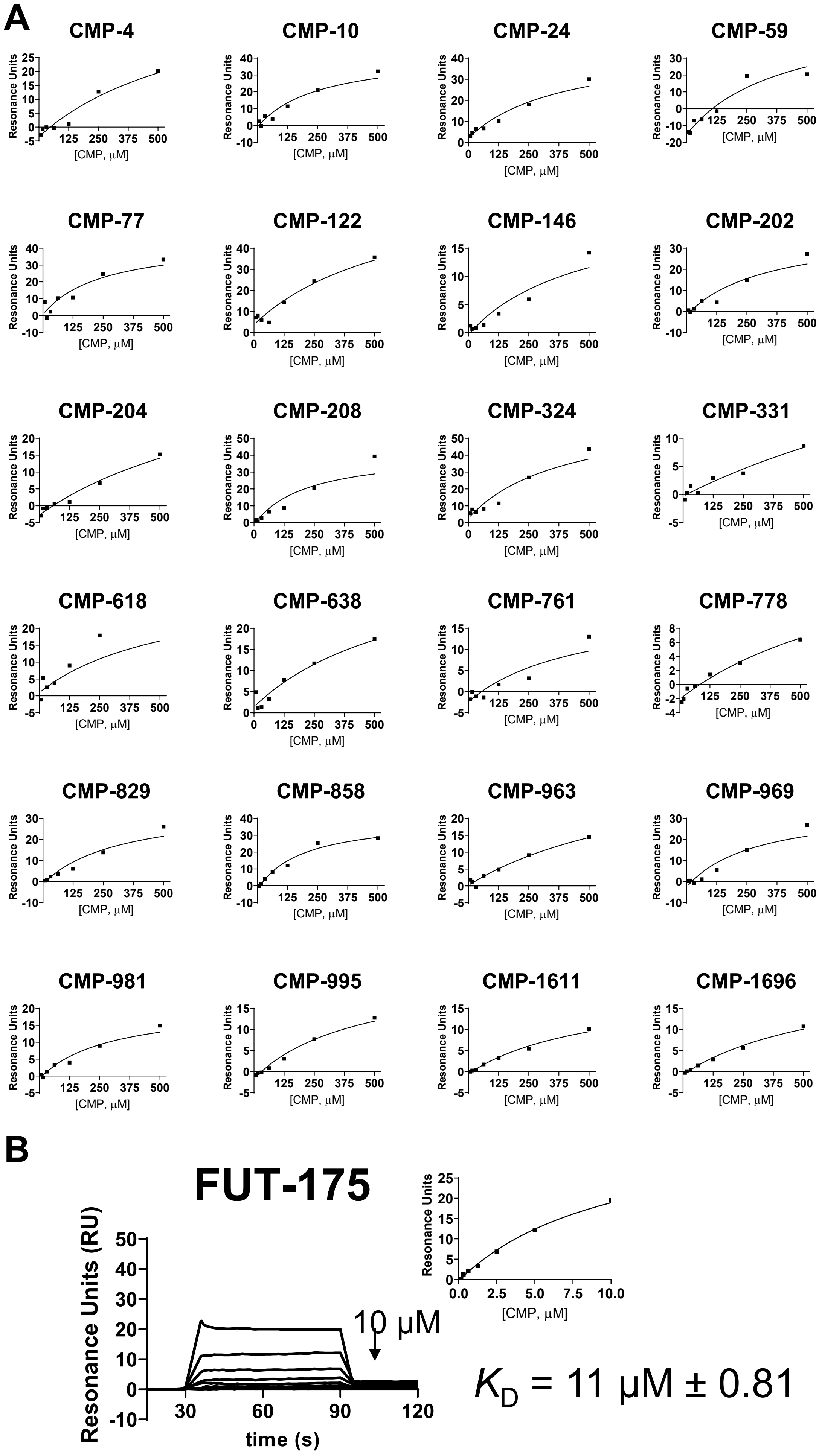

Supplementary Fig. 2

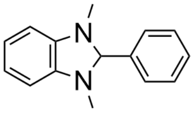

CMP-4
LE = 0.25
Mol. Wt. = 224

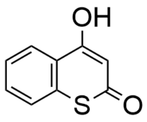

CMP-10
LE = 0.40
Mol. Wt. = 178

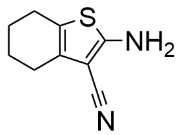

CMP-24
LE = 0.37
Mol. Wt. = 178

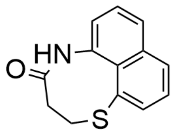

CMP-59
LE = 0.28
Mol. Wt. = 229

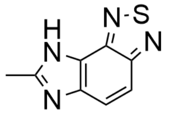

CMP-77
LE = 0.39
Mol. Wt. = 190

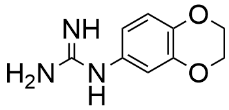

CMP-122
LE = 0.31
Mol. Wt. = 193

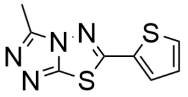

CMP-146
LE = 0.32
Mol. Wt. = 222

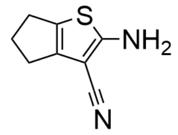

CMP-202
LE = 0.41
Mol. Wt. = 164

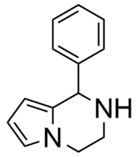

CMP-204
LE = 0.27
Mol. Wt. = 198

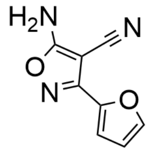

CMP-208
LE = 0.37
Mol. Wt. = 175

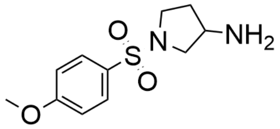

CMP-324
LE = 0.27
Mol. Wt. = 256

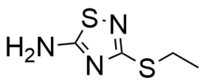

CMP-331
LE = 0.41
Mol. Wt. = 161

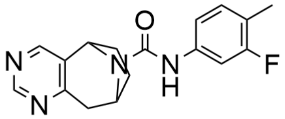

CMP-618
LE = 0.19
Mol. Wt. = 312

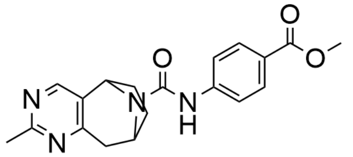

CMP-638
LE = 0.16
Mol. Wt. = 352

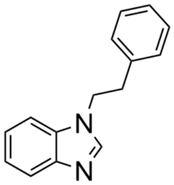

CMP-761
LE = 0.26
Mol. Wt. = 222

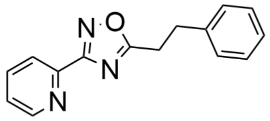

CMP-778
LE = 0.21
Mol. Wt. = 251

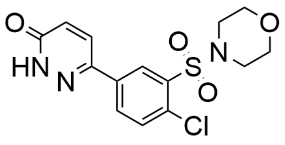

CMP-829
LE = 0.20
Mol. Wt. = 356

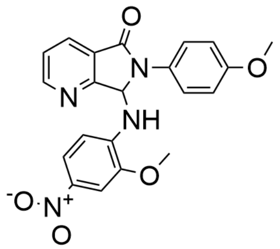

CMP-858
LE = 0.17
Mol. Wt. = 406

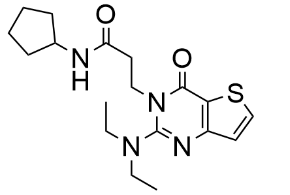

CMP-963
LE = 0.16
Mol. Wt. = 363

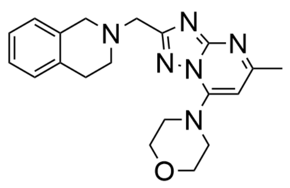

CMP-969
LE = 0.17
Mol. Wt. = 364

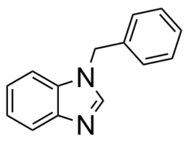

CMP-981
LE = 0.30
Mol. Wt. = 208

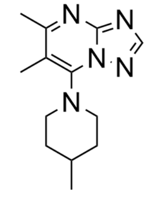

CMP-995
LE = 0.26
Mol. Wt. = 245

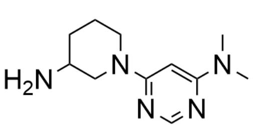

CMP-1611
LE = 0.28
Mol. Wt. = 221

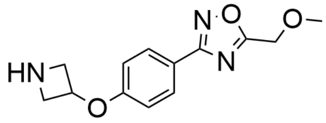

CMP-1696
LE = 0.22
Mol. Wt. = 298

Supplementary Fig. 3

A Molecular Docking onto the CUB1-EGF-CUB2 Crystal Structure (PDB:6F39)

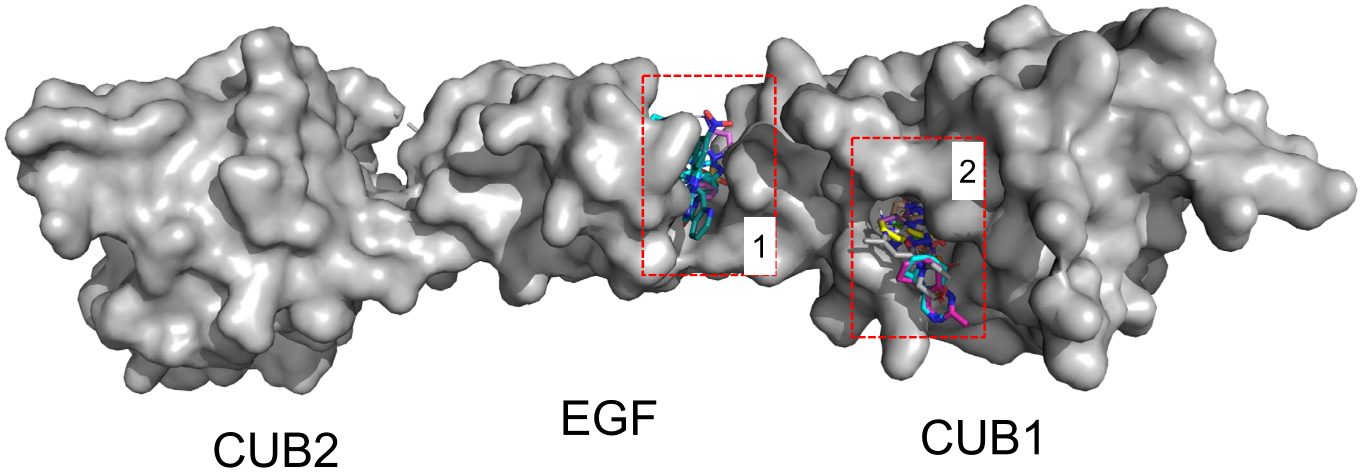

B Molecular Docking onto the C1r-CCP1-CCP2-SP Crystal Structure (PDB:1GPZ)

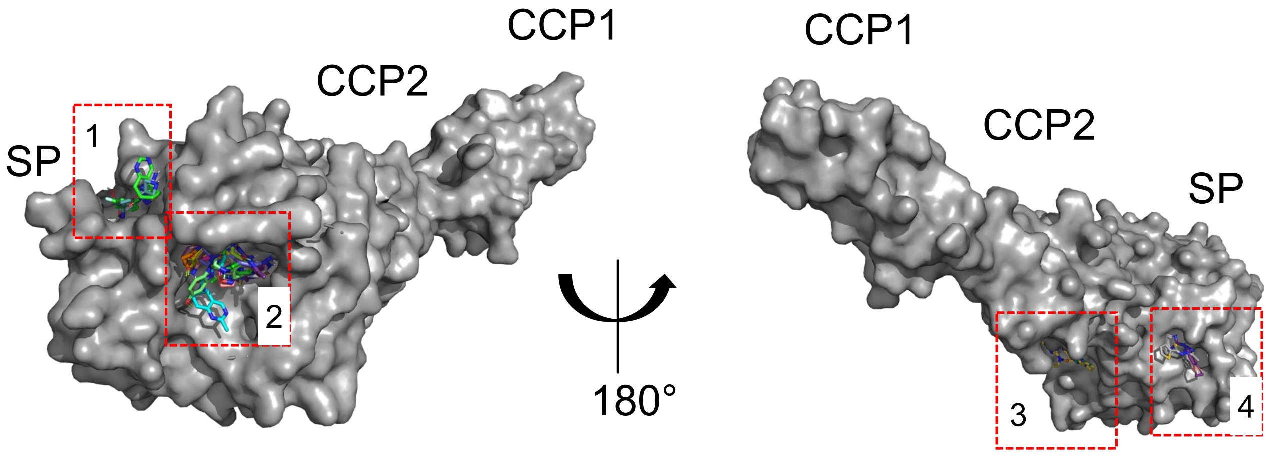

Supplementary Fig. 4

A

```
C1r       CTAQGIWKNEQKGEKIPRCLPVCGKPVNPVEQRQRIIGGQKAKMGNFPWQVFTNIHGRGG   488
C1s       CAGNGSWVNEVLGPELPKCVPVCGVPREPFEEKQRIIGGSDADIKNFPWQVFFDNP-WAG   461
MASP-2    CEADGFWTSSKGEKSLPVCEPVCGLSART--TGGRIYGGQKAKPGDFPWQVLILGGTTAA   469
          * .:* * ..    .:* * ****   .      ** **..*.  :*****:      ..

C1r       GALLGDRWILTAAHTLYPKEHEAQSNASLDVFLGHTNVEEL--MKLGNHPIRRVSVHPDY   546
C1s       GALINEYWVLTAAHVVEGNRE-------PTMYVGSTSVQTSRLAKSKMLTPEHVFIHPGW   514
MASP-2    GALLYDNWVLTAAHAVYEQKHDASA---LDIRMGTLKRLS---PHYTQAWSEAVFIHEGY   523
          ***: : *:*****.:  :..         : :*  .       :      . * :* .:

C1r       R----QDESYNFEGDIALLELENSVTLGPNLLPICLPDNDTFYDL--GLMGYVSGFGVME   600
C1s       KLLAVPEGRTNFDNDIALVRLKDPVKMGPTVSPICLPGTSSDYNLMDGDLGLISGWGRTE   574
MASP-2    T------HDAGFDNDIALIKLNNKVVINSNITPICLPRKEAESFMRTDDIGTASGWGLTQ   577
                    .*:.****:.*:: * :. .: ***** ..:   :  . :*  **:*  :

C1r       EK-IAHDLRFVRLPVANPQACENWLRGK----NRMDVFSQNMFCAGHPSLKQDACQGDSG   655
C1s       KRDRAVRLKAARLPVAPLRKCKEVKVEKPTADAEAYVFTPNMICAGGE-KGMDSCKGDSG   633
MASP-2    RGFLARNLMYVDIPIVDHQKCTAAYEKPPYP---RGSVTANMLCAGLESGGKDSCRGDSG   634
          .   *  *  . :*:.  : *                .: **:***      *:*:****

C1r       GVFAVRDPNT-DRWVATGIVSWGIG-C--SRGYGFYTKVLNYVDWIKKEMEE-------   703
C1s       GAFAVQDPNDKTKFYAAGLVSWGPQ-C--G-TYGLYTRVKNYVDWIMKTMQENSTPRED   688
MASP-2    GALVFLDSET-ERWFVGGIVSWGSMNCGEAGQYGVYTKVINYIPWIENIISDF------   686
          *.:.. * :   :: . *:****   *  .  **.**:* **: ** : :.:
```

B Docking of CMP-1696 onto C1r (PDB:1MD8)

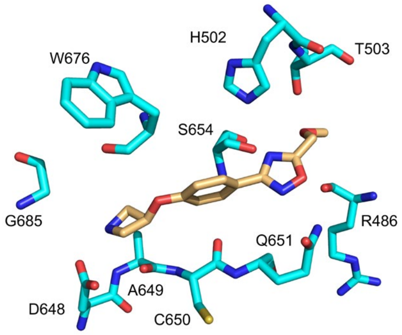

C Docking of CMP-1696 onto C1s (PDB:1ELV)

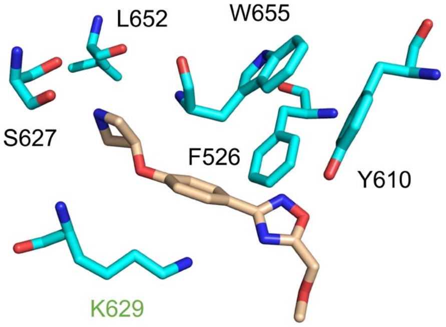

D Docking of CMP-1696 onto MASP-2 (PDB:1Q3X)

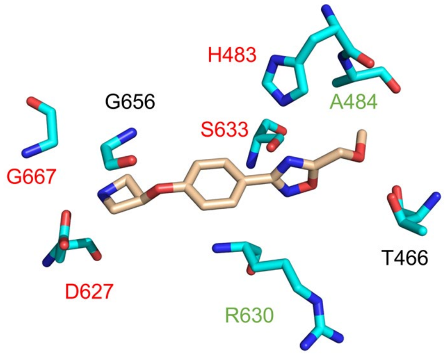

Supplementary Fig. 5

A

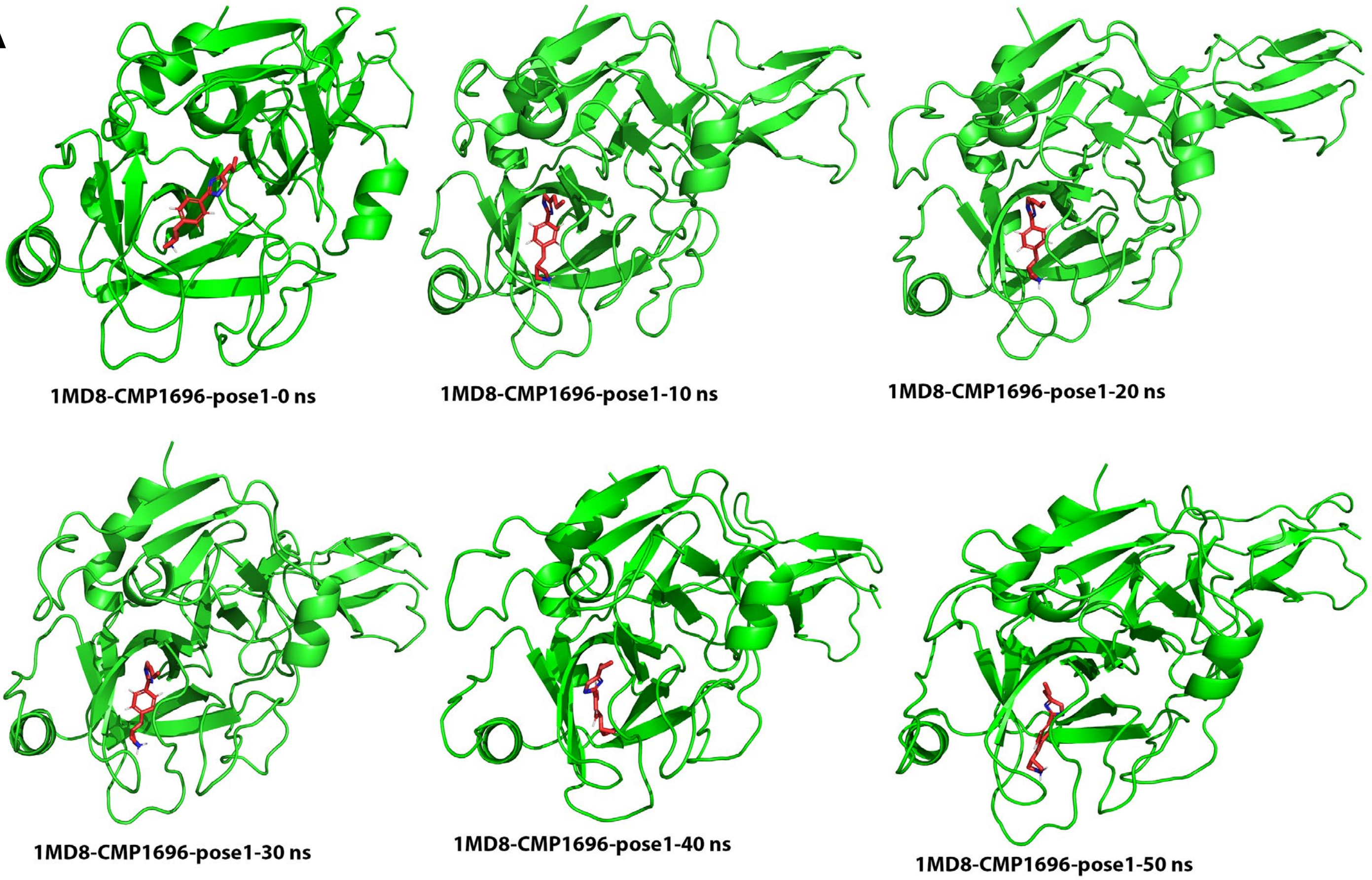

B

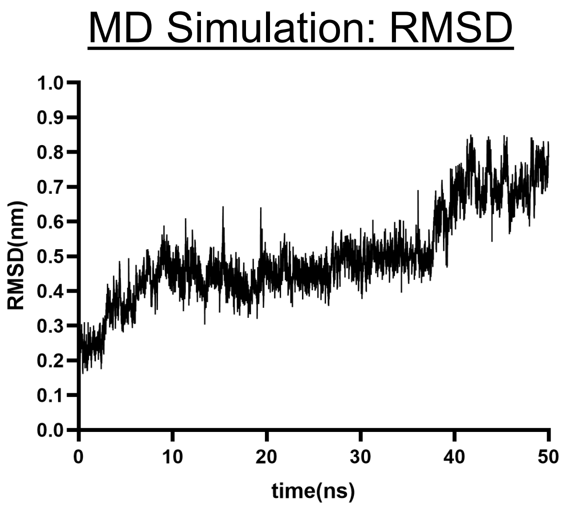

C

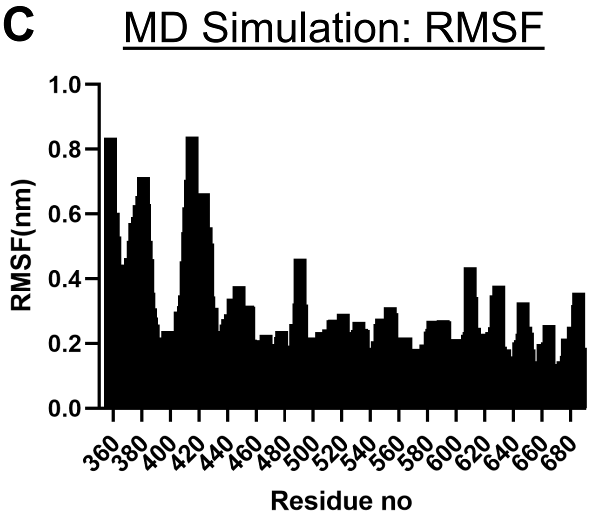

Supplementary Fig. 6

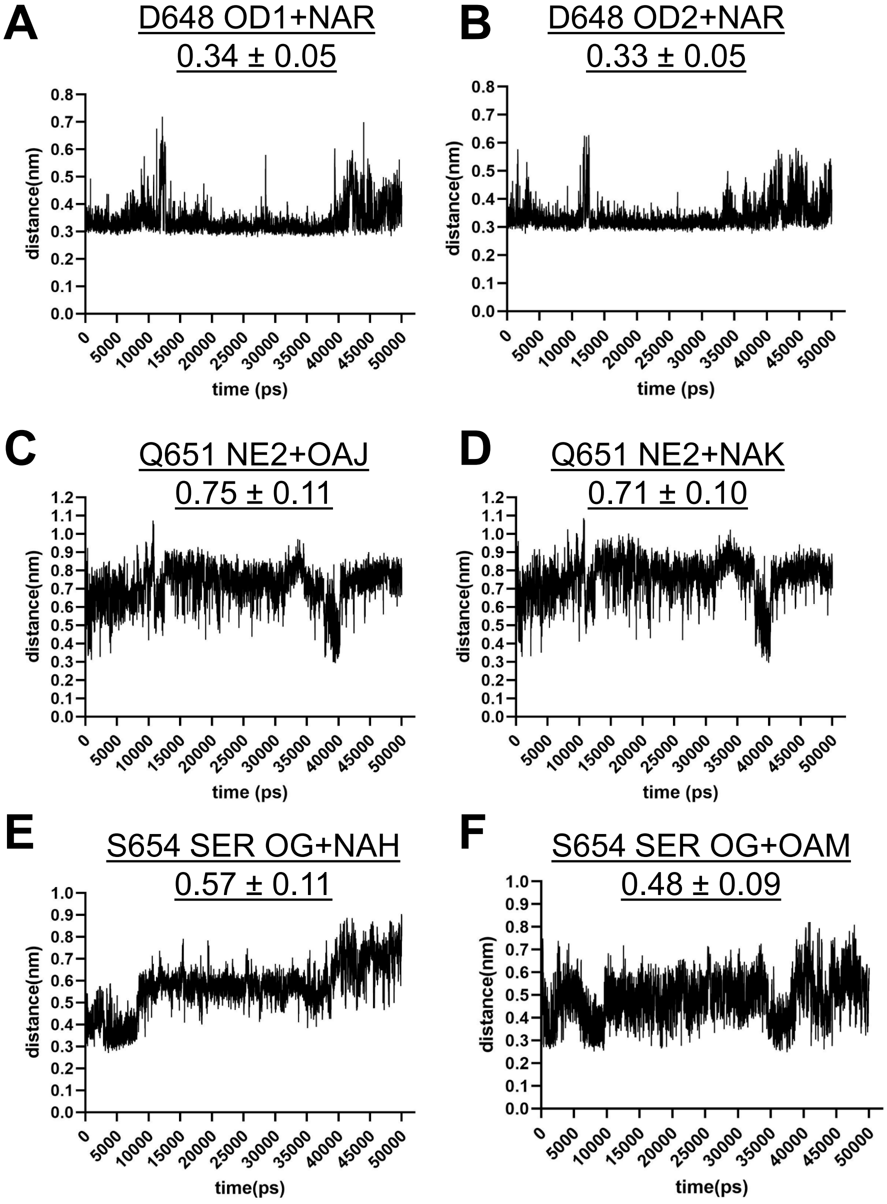

Supplementary Table 2. SPR Sensor chips used in this study

Chip	Flow-cell	Ligand	Ligand Immobilization (RU)	Experiment	Analytes†
**HC1500M-500-1**	2	C1r	17,538	Initial Screen	CMP-1 to -330
**HC1500M-500-2**	2	C1r	18,341	Initial Screen	CMP-331 to -490
**HC1500M-500-3**	2	C1r	20,251	Initial Screen	CMP-491 to -990
**HC1500M-500-4**	2	C1r	19,325	Initial Screen	CMP-991 to -1400
**HC1500M-500-5**	2	C1r	27,383	Initial Screen	CMP-1401 to -1640
**HC1500M-500-6**	2	C1r	25,268	Initial Screen	CMP-1641 to -2000
**HC1500M-DR-1**	2	C1r	29,821	Dose-response	Initial Hit Compounds
	3	C1r	16,612	Dose-response	Initial Hit Compounds
	4	C1r	22,663	Dose-response	Initial Hit Compounds
**HC1500M-DR-2**	2	C1r	33,961	Dose-response	Initial Hit Compounds
	3	C1r	8,837	Dose-response	Initial Hit Compounds
	4	C1r	9,618	Dose-response	Initial Hit Compounds
**HC1500M-DR-3**	2	C1r	8,576	Dose-response	Primary Hit Compounds
**HC1500M-DR-4**	2	C1r-CUB1	1,506	Domain Mapping	Primary Hit Compounds
	3	C1r-CCP2-SP	4,060	Domain Mapping	Primary Hit Compounds
	4	C1r	6,680	Domain Mapping	Primary Hit Compounds

† "Initial screen" refers to the single dose screen involving all 2,000 compounds, "Initial Hit Compounds" refers to the initial 95 triaged compounds, "Primary Hit Compounds" refers to CMP-1611 and CMP-1696, "Domain Mapping" refers to the data reported in Fig. 5E.
